# PReS-FINAL-2267: Successful treatment of pulmonary arterial hypertension associated with connective tissue disease (PAH-CTD) with combination therapy of sildenafil and ambrisentan: a case report

**DOI:** 10.1186/1546-0096-11-S2-P257

**Published:** 2013-12-05

**Authors:** KI Yamaguchi, M Kishimoto, M Okada

**Affiliations:** 1 Division of Allergy and Rheumatology, St. Luke's International Hospital, Tokyo, Japan

## Introduction

Patients with pediatric rheumatic disease rarely develop pulmonary arterial hypertension (PAH), and the expected prognosis had been very poor. With the advancement of oral medicine for PAH in recent years, we can expect better prognosis of these patients. For this purpose, early diagnosis and interventions are essential.

## Objectives

We report on a 14 years old girl suffering from PAH with overlap syndrome (SLE and systemic sclerosis localised type).

## Methods

At age 7, she developed autoimmune hepatitis. She was diagnosed with lupus nephritis (class II + V) from pathological finding and pulmonary hypertension (PH) at age 10, and treated with immunosuppressive therapy (methylprednisolone pulse therapy, cyclophosphamide pulse therapy and mycophenolate mofetil) and home oxygen therapy at night. Comprehensive examination about PH was carried out at age 12.

## Results

In ultra sound, tricuspid regurgitation and increased pressure gap of tricuspid valve are observed and estimated right ventricular systolic pressure was 60 mmHg. In right heart catheterization, mean pulmonary artery pressure at rest was 43 mmHg and pulmonary vascular resistance was 711 dyne·sec·10^-5^. We had diagnosed her as overlap syndrome (SLE and systemic sclerosis localized type) with PAH, and started combination therapy of sildenafil and ambrisentan. We confirmed the improvement of PAH by right heart catheterization ; mean pulmonary artery pressure (23 mmHg) and pulmonary vascular resistance (296 dyne·sec·10^-5^).

## Conclusion

Considering the obvious limitations of our single case report, we observed a good short term outcome of pediatric PAH-CTD. In order to obtain effects of oral medicine for PAH, it is important to start the intervention at early stage of this disease. It may be useful to plan screening tests (cardiac ultrasonography, pulmonary function test) on a regular basis for patients with pediatric rheumatic disease at high risk of developing PAH.

## Disclosure of interest

None declared.

